# A subcomponent-guided deep learning method for interpretable cancer drug response prediction

**DOI:** 10.1371/journal.pcbi.1011382

**Published:** 2023-08-21

**Authors:** Xuan Liu, Wen Zhang

**Affiliations:** College of Informatics, Huazhong Agricultural University, Wuhan, China; University of North Texas, UNITED STATES

## Abstract

Accurate prediction of cancer drug response (CDR) is a longstanding challenge in modern oncology that underpins personalized treatment. Current computational methods implement CDR prediction by modeling responses between entire drugs and cell lines, without the consideration that response outcomes may primarily attribute to a few finer-level ‘subcomponents’, such as privileged substructures of the drug or gene signatures of the cancer cell, thus producing predictions that are hard to explain. Herein, we present SubCDR, a subcomponent-guided deep learning method for interpretable CDR prediction, to recognize the most relevant subcomponents driving response outcomes. Technically, SubCDR is built upon a line of deep neural networks that enables a set of functional subcomponents to be extracted from each drug and cell line profile, and breaks the CDR prediction down to identifying pairwise interactions between subcomponents. Such a subcomponent interaction form can offer a traceable path to explicitly indicate which subcomponents contribute more to the response outcome. We verify the superiority of SubCDR over state-of-the-art CDR prediction methods through extensive computational experiments on the GDSC dataset. Crucially, we found many predicted cases that demonstrate the strength of SubCDR in finding the key subcomponents driving responses and exploiting these subcomponents to discover new therapeutic drugs. These results suggest that SubCDR will be highly useful for biomedical researchers, particularly in anti-cancer drug design.

## Author summary

The ability to predict the response of a cancer patient to a therapeutic drug is a major goal in modern oncology. Computational methods have led to significant progress in predicting cancer drug response (CDR), but they ignore that response outcomes may be highly associated with specific ‘subcomponents’, such as privileged substructures of drugs or gene signatures of cancer cells. To understand the effect of subcomponents in CDR prediction, we present a subcomponents-guided deep learning method, named SubCDR. SubCDR is based on multiple deep neural networks capable of extracting functional subcomponents from the drug SMILES and cell line transcriptome, and decomposing the response prediction between the drug and cell line into identifying pairwise interactions between their respective subcomponents. With this interaction form, we can obtain a traceable path to explicitly identify subcomponents contributing significantly to the response outcome. We validate our method on the GDSC dataset and demonstrate improvements over the state-of-the-art CDR prediction methods. Furthermore, we show the strength of SubCDR in interpreting the CDRs and discovering new drugs with sensitive responses from the view of subcomponent interaction.

## 1 Introduction

Precise identification of drug response in cancers holds great promise for developing personalized therapy regimens to increase survival and reduce patients’ expenses. Since testing multiple drugs for a cancer patient is infeasible for practical and financial reasons [1], there is an urgent demand for computational methods that can accurately predict cancer drug response (CDR). However, current clinical patient data with drug response labels are not large enough to train an accurate model. In recent years, several pre-clinical anti-cancer drug screen projects, such as Cancer Cell Line Encyclopedia (CCLE) [2] and Genomics of Drug Sensitivity in Cancer (GDSC) [3], have provided massive amounts of drug sensitivity profiles for thousands of cancer cell lines. Thanks to these valuable resources, researchers have been able to develop efficient computational methods for CDR prediction and systematically investigated cancer biology.

Recent years have witnessed numerous computational methods proposed to predict CDRs, which intend to relate inputs (e.g., structure profiles of drugs, omics profiles of cell lines, and association information among drugs and cell lines) to desired outputs (sensitivity/resistance classifications or specific response values). The early network-based methods constructed bio-networks with cell lines, drugs, and their known associations, and then converted the problem to a link prediction task, undertaken on the random walk [4] or information flow [5]. Matrix factorization-based methods realized CDR prediction by reconstructing the adjacency matrix of known CDRs through the product of decomposed factors [6, 7]. Traditional machine learning-based methods assembled handcrafted features capable of representing cell lines and drugs to train a classifier or regressor, including support vector machine [8] and random forest [9]. Nowadays, deep learning (DL)-based methods have achieved popularity due to their capability for providing an end-to-end solution from the feature extraction of cell lines and drugs to prediction, of which convolutional neural network (CNN)-based [10, 11] and graph neural network (GNN)-based [12–14] models exhibited powerful representational abilities. These computational methods have led to remarkable success in predicting CDRs, but most are so-called ‘black boxes’ in which the reasoning behind the prediction is unknown. It is undesirable in cancer therapy as clinicians require an explanation of why a drug is expected to work for a patient. To improve interpretability, a few efforts [15–18] focused on incorporating signalling pathway knowledge in the structures of DL-based methods so that predictions can be traced back to specific pathway nodes in the real biological network, like gene ontology (GO) [19]. And their explanations, such as DRPreter [18], were mostly oriented toward pathway signatures related to cancer cell lines.

In the context of CDR prediction, the response outcome or bio-activity is not only signalling pathway-related but may also be highly associated with the finer-level ‘subcomponents’, such as privileged substructures of drug molecules and gene signatures of cancer cells [20–23]. For example, the substructure benzodiazepine scaffold in the anti-cancer drug Devazepide is active against opioid receptors and other protein targets [24]. The varied expression status and regulation function of the tumour suppressor genes (TSGs) in different cancers plays a major role in Paclitaxel resistance [25]. Obviously, modeling the effect of drug/cell line subcomponents in CDRs holds enormous promise to uncover the relevant determinants driving response outcomes and explain the predictions, thereby enabling the discovery and design of new therapeutic drugs to overcome chemotherapy tolerance. Unfortunately, existing method frameworks for CDR prediction focus merely on modeling at the entire drug-level and cell line-level, preventing them from exploring response behaviours between drugs and cell lines at a finer-level. Hence, a pivotal challenge in CDR predictions is to develop a new interpretable computational framework that allows the model to be subcomponent-aware and provide traceable paths related to predictions. Such a framework, which additionally introduces the attention of drug signatures into modeling, has the potential to render more insights into interpretable CDR predictions compared with preceding interpretable efforts.

To address the above challenge, we propose a subcomponent-guided deep learning method for accurate and interpretable CDR prediction, named SubCDR. Instead of modeling at the entire drug (or cell line)-level, we first devise a knowledge-driven decomposition module to extract subcomponents with biological functions. For the drug (or cell line), subcomponents are depicted as a set of substructures (or gene subsets) decomposed by the molecular structure (or transcriptome profile). Afterwards, we imitate the CDR process between each drug and cell line as pairwise interactions between their respective subcomponents, and adopt a scoring function to measure the intensity of pairwise interactions, which results in an interaction map. Finally, a powerful graph convolutional network (GCN) is deployed on the interaction map to learn the representation used for decoding into the response outcome. Inspired by recommendation systems with side information [26], we also enforce the matrix factorization on a constructed response matrix of known CDR associations, to derive side information for drugs and cell lines, which can serve as potential knowledge to assist model training. Through end-to-end training, the interaction map can be viewed as a traceable path to explicitly indicate which subcomponents contribute more to the response outcome, helping to understand the model’s decisions. In the computational experiments, SubCDR outperforms other state-of-the-art CDR prediction methods on the GDSC dataset and exhibits the exclusive advantage in interpreting the predictions and discovering new drugs with sensitive responses from the view of subcomponent interaction. We believe SubCDR will be highly useful to biomedical researchers, especially in anti-cancer drug design.

## 2 Methodology

### 2.1 Data preparation

#### Response data

The cancer drug response (CDR) data were derived from the recently released large-scale drug screening datasets, named GDSC [3]. The GDSC v2 dataset comprises 135,242 instances across 190 drugs and 810 diverse human cancer cell lines, where each instance with the IC_50_ (natural log-transformed) values corresponds to a drug-cell line combination. IC_50_ [27] denotes the effectiveness of a drug in inhibiting the growth of a cancer cell line, and a small IC_50_ value reveals a high degree of drug efficacy, implying that the drug is sensitive to the corresponding cell line.

#### Cell line expression profile

Gene expression profiles are mostly used to model cancer cell lines in studying cancer biology. The cell lines project in the COSMIC database [28] provided gene expression profiles for more than 1000 cancer cell lines, where the TPM value of gene expression was log2 transformed and z-score normalized. Here, we only considered the data related to 656 cancer-driving genes from COSMIC Cancer Gene Census (CGC) portal and used them to represent the cell line.

#### Drug SMILES

The simplified molecular-input line-entry system (SMILES) is widely recognized and used as a standard profile of compounds for chemical information processing. The PubChem database [29] provided the SMILES string for each drug. We collected the drug’s SMILES according to the drug’s compound ID (CID) in the PubChem database and used it to represent the drug.

#### Pre-processing for CDR data

We processed the GDSC v2 dataset by discarding drugs that shared the same CID in PubChem and removing the cell lines for which gene expression data were unavailable from the COSMIC. Then, we excluded all the ambiguous instances, leaving only the one with the highest log-transformed IC_50_ value (i.e., ln(IC_50_)). Consequently, we compiled a dataset of 117,665 instances that measured log-transformed IC_50_ values across 800 cell lines and 175 drugs. Considering all the 800 × 175 = 140,000 drug-cell line combinations, approximately 15.95% (22,335) of all combinations’ response values were unmeasured/unknown.

### 2.2 Model architecture

**Fig 1:**
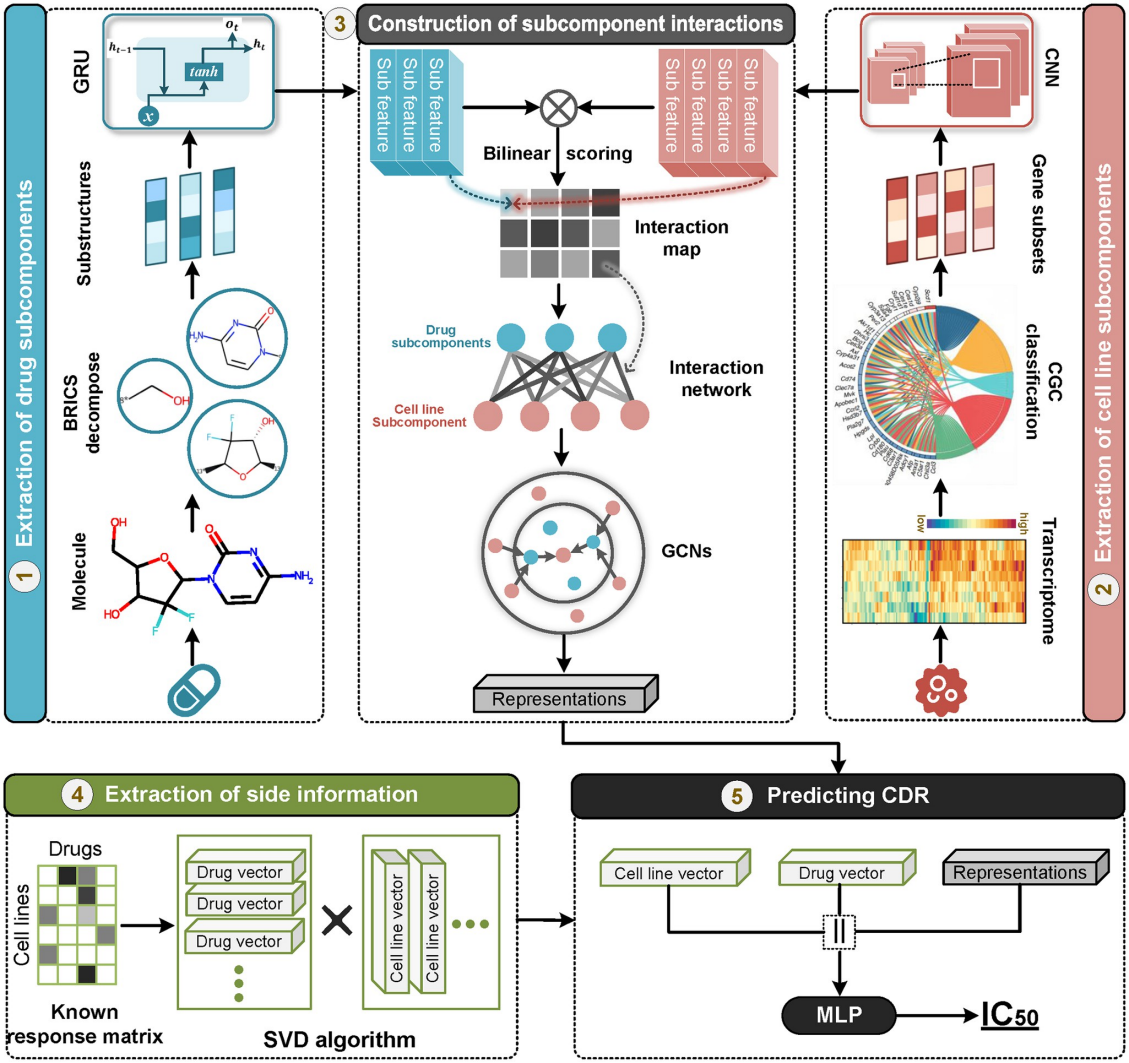
Overview of the SubCDR. (1) Extraction of drug subcomponents. The SMILES string is decomposed into a set of fragments using the BRICS algorithm, to obtain substructures (as subcomponents) for the drug, and the GRU layer is adopted to capture the latent features of substructures. (2) Extraction of cell line subcomponents. The transcriptome profile is converted into a set of gene subsets (as subcomponents) according to the CGC classification, and the latent features of gene subsets are learned by the CNN layer. (3) Construction of subcomponent interactions. An interaction map measuring interaction intensity among subcomponents is generated by Eq 5, which is further established as a network. Later, we leverage the GCN layer to learn the representations hidden in the network. (4) Extraction of side information. The side information of drugs and cell lines is acquired from the known CDRs through a singular value decomposition (SVD) algorithm. (5) Predicting CDRs. The side information combined with the learned representations is fed into a decoder, a multi-layer perceptron, to output final response values.

The overview of SubCDR is illustrated in Fig 1, which involves the following modules in an end-to-end manner. Given a drug-cell line instance, we first decompose the drug’s SMILES (or cell line’s transcriptome profile) into a set of substructures (or gene subsets) using the molecular cleavage algorithm (or CGC gene classification) and treat them as drug (or cell line) subcomponents, where each subcomponent is embedded into a latent feature via the GRU (or CNN) layer. To model the effect of subcomponents in a CDR, we break down a CDR process into pairwise interactions between subcomponents of the drug and cell line, and adopt a scoring function to measure the intensity of pairwise interactions, resulting in an interaction map. We then construct the interaction map as a network to better characterize the interaction associations among subcomponents and exploit the GCN layer to capture the representations hidden in neighborhood associations. Moreover, we extract the side information of drugs and cell lines from the known CDRs to offer potential knowledge for model training. Eventually, the side information and the learned representation are concatenated and fed into a decoder to output the predicted response value. In the following sections, we elaborate on each module and provide the details of how we operated.

#### Extraction of drug subcomponents

**Fig 2:**
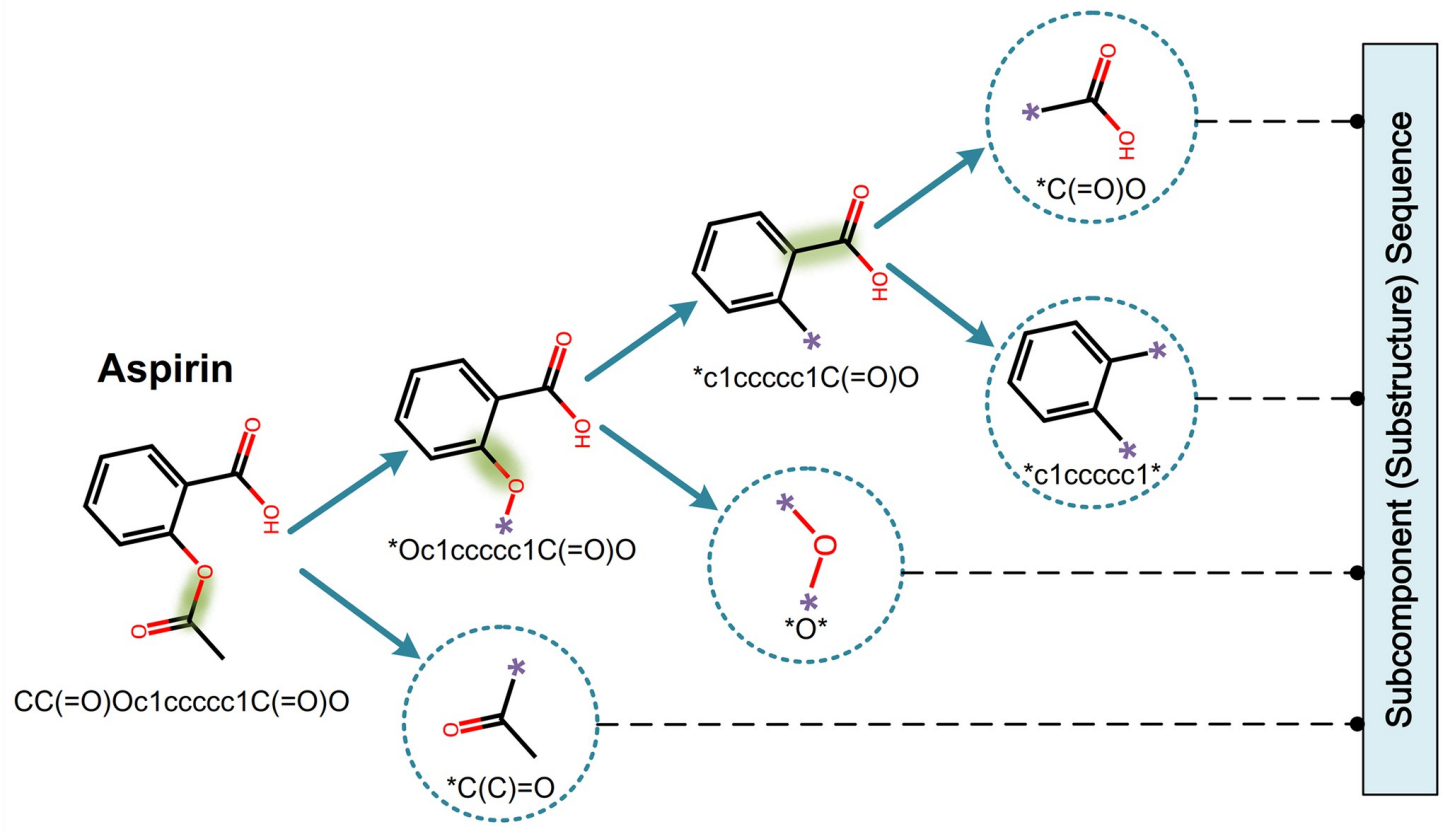
Depiction of the BRICS procedure. The root (Aspirin) of the tree is the molecule to be split, where the leaves (enclosed by dashed circles) represent the extracted substructures and ‘*’ denotes the dummy atom. At each iteration, the molecule atoms are scanned from left to right according to the SMILES order, extracting a substructure as soon as a breakable bond is found. The process is repeated until the remaining substructures cannot be split further. The dashed bonds with a green highlight are the ones chosen to break using the BRICS rules.

The Breaking of Retrosynthetically Interesting Chemical Substructures (BRICS) [30] provides a powerful algorithm to decompose molecules, which breaks strategic bonds in a molecule that matches a set of chemical reactions and retains molecular fragments with valuable substructural and functional content (e.g., aromatic rings and side-chains). Inspired by the BRICS, we decompose each drug SMILES G into an ordered sequence of substructures:

$$\begin{array}{c} \left[g_{1},g_{2},\cdots ,g_{m}\right]\leftarrow \text{BRICS}\left(G\right) \end{array}$$ (1)

where the obtained substructures are thought to be subcomponents. Take the drug Aspirin for example (Fig 2), its SMILES *CC*(= *O*)*Oc*1*ccccc*1*C*(= *O*)*O* can be divided into an ordered sequence consisting *C(*C*) = *O*, *O*, *c1*ccccc*1* and *C(= *O*)*O*, in which ‘Dummy’ atoms (‘*’) are attached to each end of the cleavage sites, marking the location where two subcomponents can join together.

For the ordered subcomponent sequence of a drug $D=[g_{1},g_{2},\cdots ,g_{m}]$, we build a recurrent neural network (RNN) module to transform it into contextual feature space. Specifically, each subcomponent *g*_*i*_ is embedded into a feature vector $d_{i}\in R^{F_{d}}$ using the extended connectivity fingerprints (ECFPs) [31]. The Gated Recurrent Unit (GRU) [32] layer is then employed to capture the contextual feature ${\hat{d}}_{i}\in R^{F}$ from the embedded subcomponent sequence:

$$\begin{array}{cc} r_{i} & =\text{Sigmoid}\;(W_{r}d_{i}+U_{r}{\hat{d}}_{i-1})\\ b_{i} & =\text{Sigmoid}\;(W_{b}d_{i}+U_{b}{\hat{d}}_{i-1})\\ o_{i} & =\text{Tanh}\;(W_{h}d_{i}+U_{\hat{d}}(r_{i}⊙{\hat{d}}_{i-1}))\\ {\hat{d}}_{i} & =b_{i}⊙{\hat{d}}_{i-1}+(1-b_{i})⊙o_{i} \end{array}$$ (2)

where ${\hat{d}}_{0}$ is the zero vector, *r*_*i*_ is a reset gate vector, *b*_*i*_ is an update gate vector, *W* and *U* are weight matrices, Sigmoid(⋅) and Tanh(⋅) stand for activation functions, *o*_*i*_ is a new hidden state, and ⊙ denotes element-wise multiplication. Because sequence lengths (i.e., number of subcomponents) decomposed by each drug could be different, subcomponent sequences of all drugs need to be fixed to a maximum length *t*_*d*_ to meet the input requirement of the GRU. For sequences less than the maximum in length, zero-padding is performed.

#### Extraction of cell line subcomponents

**Fig 3:**
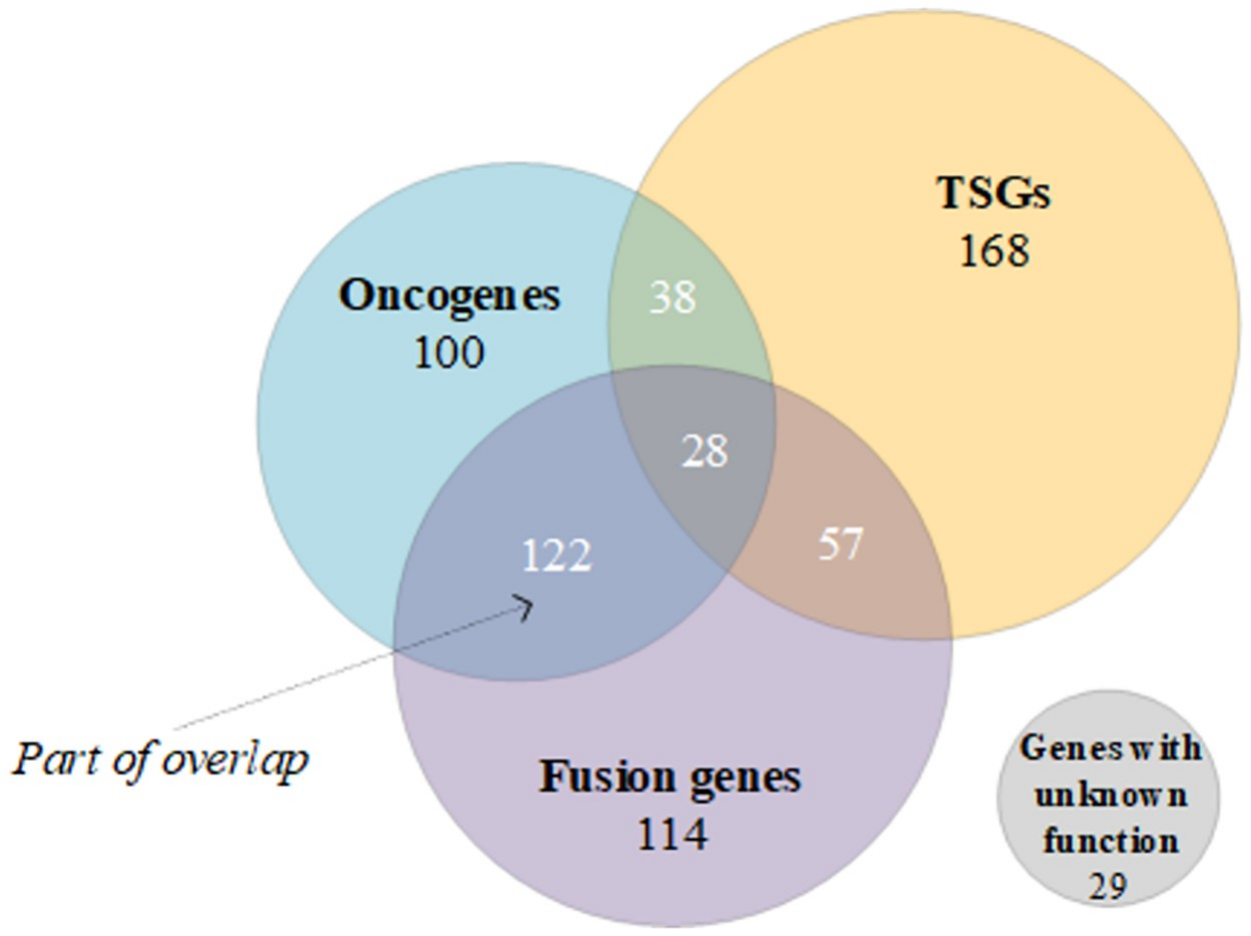
CGC gene classification. All 656 CGC genes in our work are initially divided into eight gene subsets (i.e., oncogene, tumour suppressor gene (TSG), fusion gene, the gene with unknown function (none), and their four overlaps) according to their role in cancer. Numbers correspond to the number of genes in each of the gene subsets. Note that the specific classification of CGC genes in cell lines differs, depending on the tumour type.

The COSMIC database’s Cancer Gene Census (CGC) provides mass annotations for hundreds of cancer-driving genes (CGC genes). Each CGC gene has been classified across four categories (oncogene, tumour suppressor gene, fusion gene, and gene with unknown function) depending on its somatic mutation profile and functional role in oncogenesis [33]. There is substantial overlap among these four categories of CGC genes. Following the classification of CGC, here, we first split the CGC genes of each cell line into *n* gene subsets without overlapping (as shown in Fig 3). Then, these subsets are mapped into feature vectors using the collected gene expression data, denoted as $R=\{s_{1},s_{2},\cdots ,s_{n}\}$. Given that each CGC gene in COSMIC is also annotated with the tumour types it works on, here we retain and use only the relevant genes (the tumour-specific portion) for each cell line by matching the tumor type, and the number of relevant genes varies in different tumour types (see S1(a) Text for details). Technically, we adopt a [0,1] gene masking filter (i.e., a 656-dimensional binary vector) for each cell line, where ‘1’ stands for relevant genes and ‘0’ stands for the rest. By multiplying the masking filter with the gene expression vectors R, each cell line is assured that only those CGC genes corresponding to its tumour type are involved in the downstream calculation:

$$\begin{array}{c} {\left\{c_{1},c_{2},\cdots ,c_{n}\right\}}_{j}\leftarrow {\text{MASK}}_{j}\cdot R_{j} \end{array}$$ (3)

where MASK_*j*_ is the gene masking filter of cell line *j*, and each masked gene subset expression (*c*) is regarded as a subcomponent. Different from the drug molecular substructures, gene subsets have no structural connections.

For the subcomponent set of a cell line $C={\left\{c_{i}\right\}}_{i=1}^{n}$, we implement a simple CNN to transform it into latent feature space. Specifically, since each subcomponent consists of a different number of genes, we first perform zero-padding on subcomponents whose number of genes is less than a maximum *t*_*c*_, such that the features of all subcomponents meet the same dimension. After that, we have the feature matrix $C\in R^{n\times t_{c}}$ by concatenating all subcomponents by row, and then utilize a 1D CNN with *n* channels to capture the latent features $\hat{C}$ from the feature matrix:

$$\begin{array}{c} \hat{C}\in R^{n\times F}\leftarrow \text{Conv1D}\left(C\right) \end{array}$$ (4)

where ${\hat{c}}_{i}=\hat{C}\left[i,:\right]$ is the latent feature of subcomponent *i*. Of note, the latent features of cell line subcomponents are dimensionally consistent with drug subcomponents.

#### Construction of subcomponent interactions

To measure the pairwise interactions between each subcomponent $\hat{d}$ in drug and each subcomponent $\hat{c}$ in cell line, we devise an interaction function T with a simple bilinear scoring:

$$\begin{array}{c} T\left(\hat{d},\hat{c}\right)=\text{Sigmoid}\;\left(\hat{d}\omega {\hat{c}}^{T}\right) \end{array}$$ (5)

where $\omega \in R^{F\times F}$ represents a trainable parameter matrix. The function T output is a scalar (interaction score) with a range of [0 to 1] that explicitly indicates the intensity of individual subcomponent interaction. On that basis, the intensity of all subcomponent interactions in a drug-cell line instance can be characterized as a two-dimensional interaction map $\Omega \in R^{m\times n}$. Through end-to-end learning, if a pair of subcomponents significantly attribute to the prediction, they will be updated in the downstream layer and assigned a higher score in the corresponding position of Ω. The trained interaction map can be considered a traceable path that provides hints on which subcomponents lead to the response outcome.

As the neighboring subcomponents may influence each other in triggering the interactions, the associations among subcomponent interactions should also be considered. Motivated by the power of graph convolutions, we formalize Ω as a complete bipartite network/graph G=(V,E,W,X), where V represents the set of nodes that correspond to two entities (cell line’s subcomponents $V_{C}$ and drug’s subcomponents $V_{D}$), E∈V×V denotes the set of edges indicating all possible interactions between two entities, W stores the weights (i.e., interaction scores in Ω) corresponding to edges, and $X\in R^{|V|\times F}$ is the node attributions initialized by one-hot encoding. Then we leverage the graph convolutional network (GCN) [34] layer *f*_*g*_ to the interaction network *G* so that the associations among all interactions can be captured and aggregated. Concretely, the GCN computes the hidden embedding *z* of each node by iteratively convolving over neighbour nodes using the following propagation rule.

$$\begin{array}{c} z_{v}^{\left(k\right)}=\sigma^{\left(k\right)}(\sum_{u\in N(v)\cup \{v\}}\frac{a_{vu}}{\sqrt{q_{v}q_{u}}}\cdot \Theta^{\left(k\right)}\cdot z_{u}^{\left(k-1\right)}) \end{array}$$ (6)

where $z_{v}^{\left(k\right)}=Z^{\left(k\right)}\left[v,:\right]$ stands for the embedding of node *v* in the *k*-th layer with $Z^{\left(0\right)}=X$; *σ* is the activation function using LeakyRelu; N(v) denotes a set of nodes adjacent to *v* in E; $q_{v}=1+\left|N\left(v\right)\right|$; Θ is the learnable matrix parameter; $a_{vu}\in W$ denotes the edge weight from node *v* to node *u*. After *K* GCN layers, we have the learned embeddings of all nodes in a network: *Z*^(*K*)^.

Next, we apply a global pooling layer over the learned embeddings of all nodes *Z*^(*K*)^ to produce a summary representation *h* for the entire interaction network:

$$\begin{array}{c} h\in {\mathbb{R}}^{F^{\prime }}\leftarrow [{\text{GM}}_{\text{ax}}\text{P}\left(Z^{\left(K\right)}\right)\;\|\;{\text{GM}}_{\text{ean}}\text{P}\left(Z^{\left(K\right)}\right)] \end{array}$$ (7)

where GM_ax_P/GM_ean_P is the global max/mean pooling layer and ∥ denotes a vector concatenation operator.

#### Extraction of side information

Apart from the above biochemical features of subcomponents, we expect to mine more knowledge to guide model training. Side information, in artificial intelligence, is data that comes from neither the input space nor the output space [26], and its purpose is to enrich input space with the aid of other available potential knowledge. One effective way to obtain side information for drugs and cell lines is to extract association information hidden in known CDRs. In detail, we define a response matrix $R\in R^{|V_{D}\left|\times \right|V_{C}|}$, where *R*_*ij*_ = *Null* if no measured response value between the drug $i\in V_{D}$ and the cell line $j\in V_{C}$ in the training set; otherwise *R*_*ij*_ is equal to their corresponding log-transformed IC_50_ value. Subsequently, we utilize the singular value decomposition (SVD) [35] to decompose the response matrix into two low-rank matrices (*I* and *J*), which serve as side information for drugs and cell lines. The objective of SVD to be minimized is as follows:

$$\begin{array}{c} \underset{I,J}{\text{min}}\;\frac{1}{2}{\left|M⊙(R-I^{T}J)\right|}_{F}^{2} \end{array}$$ (8)

*M* is a masked matrix where *M*_*ij*_ = 1 if *R*_*ij*_ is a measured response value; otherwise *M*_*ij*_ = 0. | ⋅ |_*F*_ stands for the Frobenius norm. Benefiting from the matrix factorization paradigm, *I* and *J* are latent factors for the response matrix and contain potential knowledge representing known CDR associations, of which *I*[*i*, :]/*J*[*j*, :] indicates side information about the *i*/*j*-th drug/cell line. Further, we feed the *I* and *J* into a fully-connected layer *f*_*a*_ to output their latent vectors as the final side information ($\hat{I}$ and $\hat{J}$), like the subcomponent extraction pipeline.

#### Predicting CDRs

To output the final response outcome for a drug-cell line instance (*i*, *j*), we design a commonly used decoder from the learned representation (*h*_*ij*_) and side information ($\hat{I}\left[i,:\right],\hat{J}\left[j,:\right]$) to the log-transformed IC_50_ value $\hat{p}$:

$$\begin{array}{c} {\hat{p}}_{ij}=\text{MLP}(h_{ij}\left\|\hat{I}\left[i,:\right]\right\|\hat{J}\left[j,:\right]) \end{array}$$ (9)

where MLP is a multi-layer perceptron. The huber loss [36], a combination of the mean squared error (MSE) and the mean absolute error (MAE) used in robust regression, is adopted as our objective function, formulated as:

$$\begin{array}{cc} L & =\frac{1}{\left|S\right|}\sum_{(i,j)\in S}L_{ij}\\ L_{ij} & =\{\begin{array}{cc} \frac{1}{2}{\left({\hat{p}}_{ij}-p_{ij}\right)}^{2} & |{\hat{p}}_{ij}-p_{ij}|\leq \delta \\ \delta |{\hat{p}}_{ij}-p_{ij}|-\frac{1}{2}\delta^{2} & \text{otherwise} \end{array} \end{array}$$ (10)

where S is the training set of CDR instances, *p*_*ij*_ denotes the true value of the response between the drug *i* and the cell line *j*, and *δ* is a scale parameter that defines the boundary where the loss function transitions from quadratic to linear.

#### Setup

The hyperparameters of SubCDR are recommended as follows. In the extraction of drug subcomponents, we set the radius and fixed length of ECFPs to 2 and 512, respectively; the maximum length *t*_*d*_ is determined by the drug that decomposes the largest number of molecular substructures; the number of GRU layers is set to 2. In the extraction of cell line subcomponents, the maximum *t*_*c*_ is determined by the cell line subcomponent with the largest number of genes, and the number of CNN layers is set to 2. We select a 2-layer simplifying GCN for *f*_*g*_ to update node embeddings in handling the interaction network. The *f*_*a*_ in the extraction of side information is a 2-layer fully-connected network with Batch normalization. In predicting CDRs, the decoder is a 3-layer MLP with Dropout, and scale parameter *δ* is fixed to 1. We use the Adam with a learning rate of 0.0001 to optimize the entire model. Detailed hyperparameter settings are listed in our source codes (https://github.com/liuxuan666/SubCDR).

### 2.3 Experiment settings

#### Performance evaluation

To comprehensively evaluate the performance of SubCDR, we randomly selected 90% instances from the dataset to compile the cross-validation set and used the remaining 10% instances as the independent test set. For the cross-validation set, we performed the following three practical scenarios:

- **Warm start**. The 5-fold cross-validation (5-CV) was implemented by randomly dividing all instances into 5 equal parts, and the training and test sets share common drugs and cell lines.
- **Cold start for cell line**. The 5-CV was implemented by randomly splitting instances on the cell lines to guarantee that the test set only includes unseen cell lines in the training set.
- **Cold start for drug**. Similar to the scenario above, the 5-CV was conducted on the drugs, where the test set only contains the drugs absent in the training set.

Additionally, we trained the model on the whole cross-validation set, and then made predictions over the independent test set for a more objective evaluation (also called independent testing). Three common metrics are used for measuring the statistical correlation between observed values and predicted response values under a regression task, encompassing Root Mean Squared Error (RMSE), Pearson’s Correlation Coefficient (PCC), and the Coefficient of determination (R^2^). To further confirm the classification accuracy of models, we binarized the log-transformed IC_50_ value in accordance with a threshold used in previous studies [37], to generate sensitive (positive) and resistant (negative) instances. Drug-cell line instances with a log-transformed IC_50_ value below -2.0 (-2.0 corresponds to IC_50_ of approximately 0.135 *μ*M) were classified as positive instances; otherwise, they were classified as negative instances. The ratio of positive and negative instances is around 1:10. We then expanded the above SubCDR framework into the classification task by adding a Sigmoid activation at the last layer and taking binary cross entropy (BCE) as the loss function. The classification performances are evaluated by two metrics: the area under curve (AUC) and the area under the precision-recall curve (AUPR).

#### Baseline methods

We evaluated our method against several state-of-the-art methods for CDR prediction.

- **tCNNs** [10] employed the CNNs to predict CDR, and the SMILES sequences of drugs and genomic mutation data of cell lines are used for the input features.
- **DeepCDR** [12] integrated multi-omics profiles of cell lines and chemical structures of drugs, and then developed a hybrid GNN to predict CDRs.
- **DrugCell** [15] developed an interpretable deep learning model with visible neural networks to make CDR predictions, which assembles genotype embedding of cancers and structure embedding of drugs.
- **GraphCDR** [13] accomplished a GNN-based framework based on expression profiles of cell lines, chemical structures of drugs, and their known responses for CDR prediction.
- **Bi-GNN** [14] predicted CDRs through a graph representation learning method that incorporates information regarding the sensitivity and resistance of cell lines.

The implementation details of the above methods are described in S1(b) Text.

## 3 Results

### 3.1 Performance comparison

**Fig 4:**
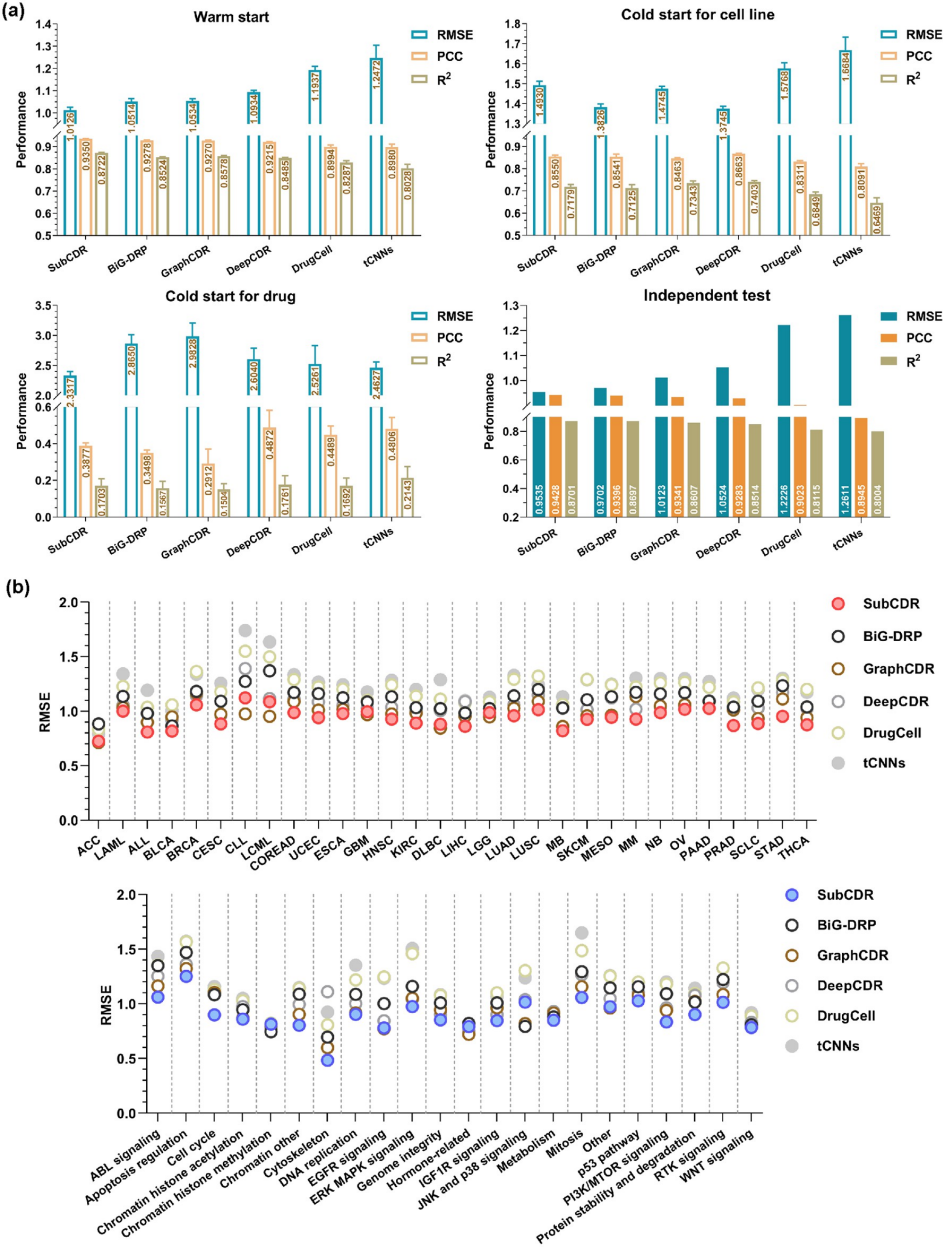
The performances of SubCDR and baseline methods. (a) RMSE, PCC, and R^2^ scores of all methods on three scenarios and the independent test. (b) RMSE scores of all methods across different cancer types of cell lines (defined in the TCGA study, up) and target pathway types of drugs (down).

The performances of SubCDR and baseline methods are shown in Fig 4a. In the warm start, SubCDR achieved lower RMSE, higher PCC and R^2^ scores of 1.0126, 0.9350 and 0.8722 than baseline methods, implying its strong capability in predicting CDRs. By contrast, the cold start for cell line and drug is more challenging, which tests model performance in predicting unseen cell lines and drugs. It is observed that the performance of all methods significantly decreased in these two scenarios. Among all methods, SubCDR produced competitive performance in the cold start for cell line but sub-optimal performance in the cold start for drug. When solving the cold start problem, the core idea is to assign the response values of seen drugs/cell lines to unseen ones if there are molecular structural/gene expression similarities. However, our subcomponent form inevitably disrupted the entire structure of the drug molecule and degraded the original characterization, which makes our model hard to transfer to unseen drugs. For another, the performance of all methods in the cold start for drug was lower than that in cell lines. One possible reason is that the number of drug types (175) in the dataset is far less than the number of cell line types (800), so the models are more difficult to get enough knowledge for migrating in unseen drugs. From the overall results of cold starts, our model can be applicable to the prediction of unseen drugs/cell lines.

From the regression performances on the independent testing (Fig 4a), SubCDR outperformed all the baselines and exceeded the best baselines: Bi-GNN and GraphCDR, by 1.75% and 6.16% in RMSE scores, 0.34% and 0.93% in PCC scores, and 3.69% and 1.60% in R^2^ scores. For the classification tasks we conducted on the independent test, the performances for all methods are shown in S1 Fig. As a result, SubCDR achieved the highest AUC and AUPR scores of 0.9885 and 0.9103 among all compared methods, validating its high generalization ability and strong predictive power in both regression and classification tasks.

In addition to the global performance of the methods, we focused on the predicted result of instances divided into different cancer and target pathway types. Each drug was annotated with a target pathway type in accordance with the GDSC database (S1(a) Table), and each cell line was annotated with a cancer type defined in The Cancer Genome Atlas (TCGA) study [38] (S1(b) Table). As seen in Fig 4b, we grouped the independent testing results according to the above two types, respectively, and then calculated their RMSE scores for assessment. Among the 32 different cancer types, SubCDR showed the highest average prediction accuracy in most cancer types and revealed a consistently high performance by achieving an RMSE score ranging from 0.7247 to 1.1230. Meanwhile, SubCDR was also shown to provide higher accuracies across 22 types of target pathways compared to other baselines. In the results for the other metrics: PCC, R^2^, AUC, and AUPR, as shown in S2 and S3 Figs, SubCDR still displayed the higher prediction accuracy in most cancer and target pathway types, and a minor variance across these types. Different cancer/target pathway types showed more significant variance on classification tasks than on regression tasks. This phenomenon may result from the imbalance ratio, i.e., the discrepancy between the number of sensitive and resistant instances, across the different types.

### 3.2 Interpreting prediction from the subcomponent interaction

**Fig 5:**
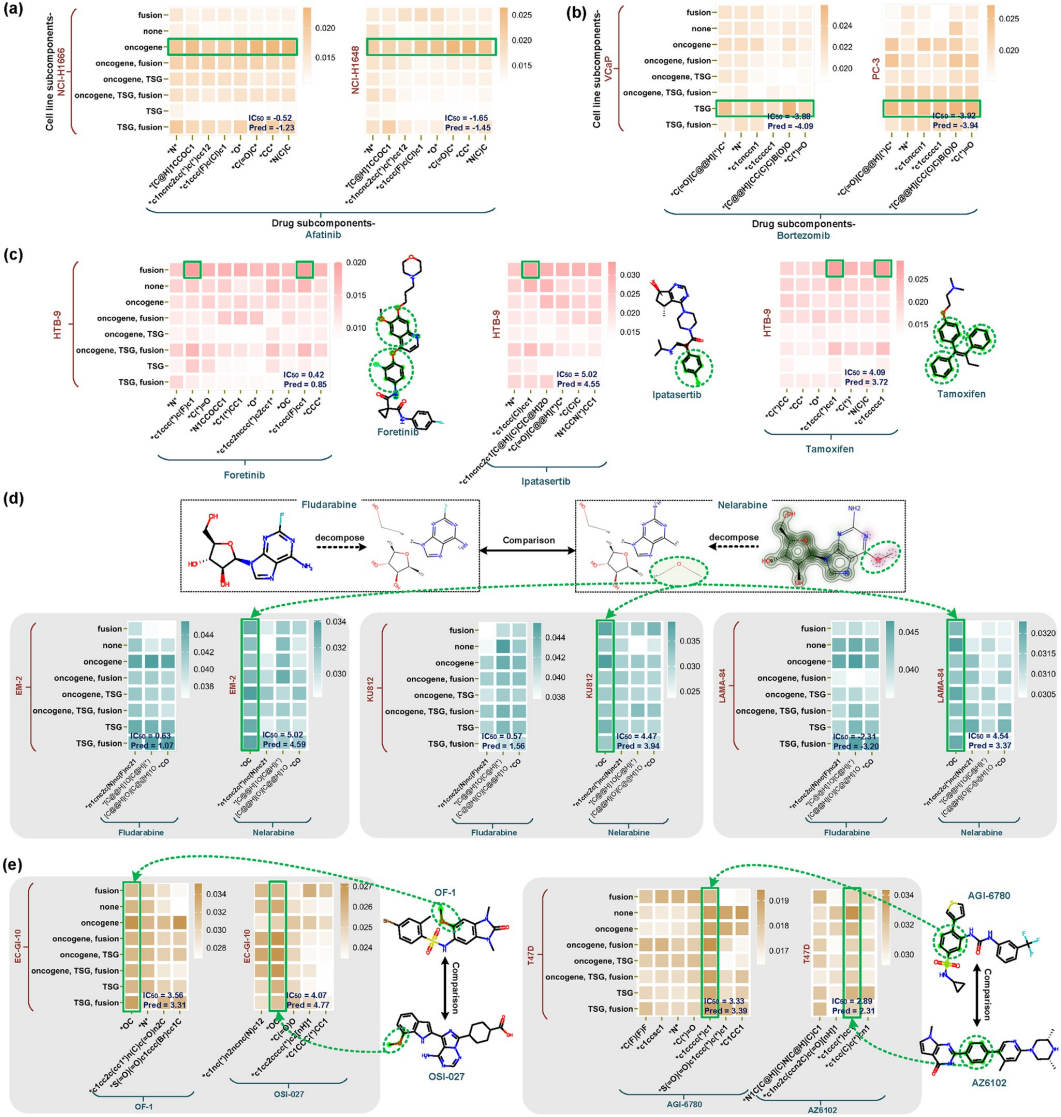
Visualization of subcomponent interactions, where each interaction map is processed by Softmax and generated as a heatmap, the rows/columns on the map denote cell line/drug subcomponents. (a) Cases of drug Afatinib’s response to cell lines NCI-H1666 and NCI-H1648. (b) Cases of drug Bortezomib’s response to cell lines VCaP and PC-3. (c) Cases of cell line HTB-9’s response to Foretinib, Ipatasertib, and Tamoxifen. (d) Cases of drug Fludarabine and Nelarabine responding to cell lines EM-2, KU812, and LAMA-84. (e) Cases of drug OF-1 and OSI-027 responding to cell line EC-GI-10, and cases of drug AGI-6780 and AZ6102 responding to cell line T47D.

In the prediction phase, SubCDR generated an interaction map scoring interaction intensities between subcomponents for an input drug-cell line instance, and subcomponents (or their interactions) that contribute significantly to the response outcome will return higher scores. Here, we visualized the interaction maps as heatmaps to highlight the subcomponent interactions with higher scores. By comparing and analyzing the heatmaps for many instances, we observed partial cases that can prove the strength of SubCDR in highlighting the critical subcomponents that influence the response outcomes and interpreting the prediction in a biologically meaningful order:

In Fig 5a, the oncogene subcomponent resulted in the highest interaction intensity in the interaction maps of Afatinib to lung carcinoma cell lines NCI-H1666 and NCI-H1648. Plenty of studies [39] also pointed out that Afatinib is an irreversible ErbB family blocker, targeting oncogenes such as EGFR, ERBB2 and ERBB3 (that are also included in the oncogene subcomponent of the two cell lines), and is widely used in the treatment of lung cancer. By observing the interaction maps in Fig 5b, TSG gene subcomponents of prostate cancer cell lines VCaP and PC-3 were found to be highly related to the sensitive responses with Bortezomib. From previous trials in prostate cancer [40], Bortezomib is a proteasome inhibitor that has an anticancer effect in cell/animal models for prostate cancer. TSG genes (e.g., TP53, AXIN1 and PTEN, in the TSG gene subcomponent of VCaP and PC-3) were known to affect the aggressiveness of prostate cancer [41], corresponding to the Wnt signaling pathway, which is also a target pathway associated with Bortezomib [42]. In Fig 5c, we found a shared phenyl group in the responses of bladder carcinoma cell line HTB-9 with drugs Foretinib, Ipatasertib, and Tamoxifen, its interaction with fusion genes had a high coefficient in their heatmaps. Beyond that, we observed that Gefitinib produces approximate interaction maps (the score distributions of two heatmaps are similar) with cell lines NCI-H1650 and NCI-H1568, both of which belong to the Non-small Cell Lung Carcinoma (NSCLC) (S4(a) Fig). Of the eight cell line subcomponents, fusion genes have been reported as a fundamental cause of NSCLC tumorigenesis [43, 44], and their related subcomponents are also highlighted in interaction maps. Analogous findings were also seen in the response of Dactolisib with lymphoblastic leukemia cells KARPAS-45 and P30-OHK (S4(b) Fig), showing that ‘closer’ cell lines may have approximate response outcomes and interaction maps with the same drug.

In addition to the explanatory cases above, we noticed that a few structurally similar drugs respond differentially to the same cell line, which subcomponent changes may cause. For example, the addition of the carbonyl group to Fludarabine (which became Nelarabine) significantly reduced the sensitivity to chronic myelogenous leukemia cell lines such as EM-2, KU812, and LAMA-84, and the carbonyl group also received the highest scores in their subcomponent maps (Fig 5d). That may be owing to the replacement of fluorine atoms, whose modified drugs have been proven to induce apoptosis in leukemia [45]. In cases of Cytarabine and Gemcitabine responding to lung adenocarcinoma cell lines HCC78, NCI-H1792, and NCI-H1993 (S4(c) Fig), their interaction maps highlighted that the difluoro group appears to be a major determinant in the occurrence of sensitive responses, and a lot of polyfluorinated compounds, e.g., Entrectinib, Gemzar, and Lumakras, are also FDA-approved for lung cancer therapy (https://www.cancer.gov/about-cancer/treatment/drugs/lung). Otherwise, a few drugs with diverse structures produced similar response outcomes on the same cell line. For example, the pan-BRPF bromodomain inhibitor OF-1 and the mTOR kinase inhibitor OSI-027 differ significantly in molecular structure, but they responded similarly in the esophageal carcinoma cell line EC-GI-10, and similar situations also occurred in the responses of drugs AGI-6780 and AZ6102 with breast invasive carcinoma cell line T47D (Fig 5e). Explaining from our subcomponent interactions, subcomponents shared by both drugs (i.e., the highlighted carboxyl group and phenyl group in Fig 5e, respectively), are probably the key to causing these situations.

### 3.3 Discovering novel CDRs leveraging subcomponent interaction

**Fig 6:**
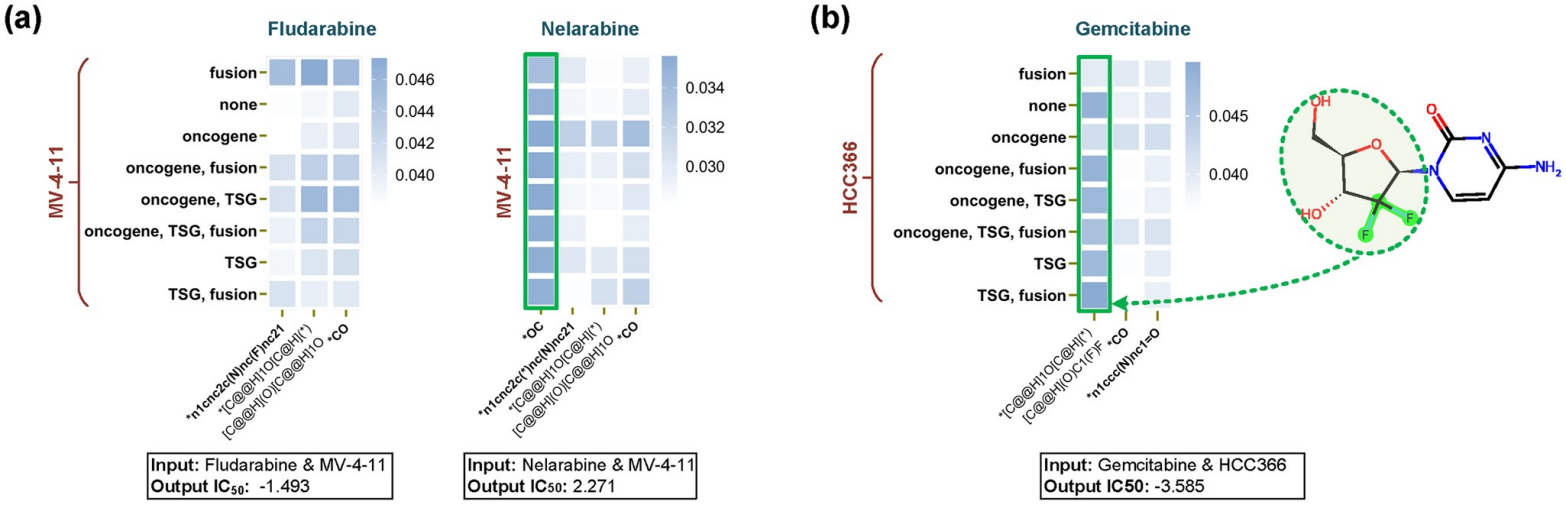
Representative examples illustrating the advantage of SubCDR in discovering new drugs with sensitive responses. (a) Cases of cell line MV-4–11’s response to drug Fludarabine and Nelarabine. (b) Case of cell line HCC366’s response to drug Gemcitabine.

As the interpretability of SubCDR underlines crucial subcomponents, here we investigate whether these subcomponents can guide the discovery of new drugs with sensitive responses. Depending on the findings in Fig 5d, we tested drugs Fludarabine and Nelarabine on a unseen leukemia cell line MV-4–11. Also, we observed that the carbonyl group leads to the switching from sensitivity (-1.493 of predicted value) to resistance (2.271 of predicted value) between these two drugs (Fig 6a), and the predicted response classifications were able to be verified in previous research [46]. Furthermore, we predicted the drug Gemcitabine’s response to a unseen NSCLC cell line HCC366 based on the findings in S4(c) Fig, its interaction map also emphasized the major impact of the difluoro group (Fig 6b), and the sensitivity outcome (-3.585 of predicted value) was supported by [47]. These cases revealed that the interpretability of SubCDR can provide valuable guidance in discovering novel anti-cancer drugs.

To further verify the ability of SubCDR to discover novel sensitive CDRs, we paid attention to the unmeasured drug-cell line combinations mentioned in Section 2.1. We trained the SubCDR using all the measured instances in the dataset and then predicted the unmeasured combinations. Here, two clinically approved drugs, Gemcitabine and Bortezomib, were taken for verification. We selected the top 10 cell lines with the lowest predicted log-transformed IC_50_ values (values also need to be less than -2) for these two drugs as the candidates that may produce sensitive responses (Table 1), and then guided a literature search and found that seven predicted CDRs were consistent with observations from previous studies. For example, according to Smith et al.’s study [48], Gemcitabine was cytotoxic to lymphoma cell line DoHH-2 in vitro, and DoHH-2 cells are growth inhibited by Gemcitabine with an IC_50_ of 1 nM. When neural stem cells H9 were treated with Gemcitabine, the measured IC_50_ was reported as only 0.0015 *μ*M (i.e., log-transformed IC_50_ = -6.502) [49] and can be identified as sensitivity by threshold provided in [3]. Many studies [50] also suggested that Gemcitabine is an approved chemotherapy drug with activity in non-small-cell lung cancer (NSCLC) such as NCI-H2135 cell line. The cell line DoHH-2 showed sensitivity to Bortezomib, and the maximum cell death induced by Bortezomib was reached in 24 hours [51]. Bortezomib was reported to reduce the growth of the melanoma cell line SK-MEL-5 with activated caspase-3 mediated apoptosis [52]. In Liew et al.’s trials [53], the proliferation of ovary adenocarcinoma cell EFO-27 and its subclone cells was inhibited after exposure to Bortezomib. Malignant pleural mesothelioma cells, such as NCI-H2803 and MSTO-211H, have been found to be sensitive to the clinically approved proteasome inhibitor Bortezomib [54]. The above evidence supported our claim that SubCDR can be useful in uncovering possibly efficacious drugs for cancer treatment.

**Table 1:** Top 10 cell lines with the lowest predicted response values of two approved drugs.

Drug	Predicted log(IC_50_)	Cell line	PMID
Gemcitabine	-5.8049	DOHH-2	16109167
	-4.6414	ALL-PO	-
	-4.6266	H9	24684846
	-3.9962	NCI-H2810	-
	-3.8506	OVK-18	-
	-3.6913	SKM-1	-
	-3.6809	CESS	-
	-3.4111	NCI-H2135	12530041
	-3.1946	G-MEL	-
	-3.0654	HuTu-80	-
Bortezomib	-6.5158	JURL-MK1	-
	-6.4762	DOHH-2	22393418
	-5.9688	GMS-10	-
	-5.8199	SK-MEL-5	24310621
	-5.6204	HuTu-80	-
	-5.5220	HCC-366	-
	-5.5197	KYSE-510	-
	-5.5072	RPMI-8866	-
	-5.5068	EFO-27	29451304
	-5.4772	NCI-H2803	33240401

### 3.4 The complete version of SubCDR demonstrates the best performance

SubCDR provides an interpretable CDR prediction framework that integrates a line of modules for handling subcomponent extraction, subcomponent interaction and side information acquisition. To investigate the necessity of each module in our model architecture, we conducted several comparisons between SubCDR with its variants:

- SubCDR without side information (w/o SI). We removed the side information learned from the known CDR matrix.
- SubCDR without GRU and CNN layer (w/o GC). We removed the GRU/CNN layer used to extract the hidden features of the drug/cell line subcomponents, and directly used ECFPs and gene expression data to calculate the interaction map.
- SubCDR without interaction network (w/o IG). We removed the interaction network and GCN layer, and then output the learned embedding by applying a flattening layer to the interaction map.
- SubCDR without GCN layer (w/o GCN). We replaced the GCN layer with a gated GNN [55] capable of handling weighted networks to learn the embeddings from our interaction networks.
- SubCDR without subcomponent parts (w/o SP). We removed the extraction and interaction modules related to drug and cell line subcomponents, and assembled the rest into a simplified prediction model with ECFP as drug input and gene expression data as cell line input.

**Fig 7:**
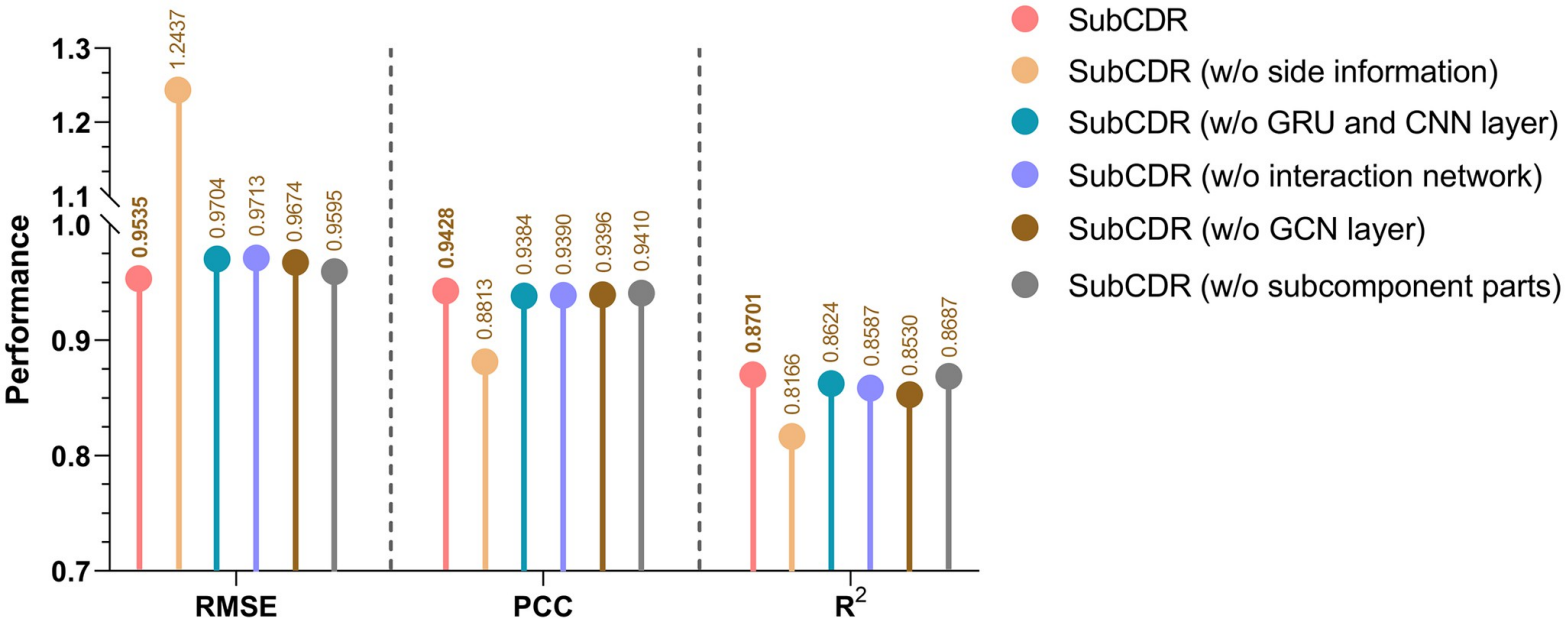
Performances of SubCDR with its variants (ablation analysis).

The comparisons were operated on the independent testing, and the results are shown in Fig 7. When the side information (w/o SI) was removed, the performance in terms of RMSE, PCC and R^2^ dropped from 0.9535 to 1.2437, 0.9428 to 0.8813, and 0.8701 to 0.8166, respectively, implying the usefulness of this potential knowledge. The result of the variant (w/o GC) showed that using the GRU/CNN layer to extract the latent features of drug/cell line subcomponents contributes to the prediction. As expected, the performance of the variant (w/o IG) signified that the prediction is boosted using the interaction network to capture the associations among subcomponent interactions. From the variant (w/o GCN) result, we observed that the GCN layer outperforms the gated GNN on CDR prediction. After removing the subcomponent parts (w/o SP), the performance of SubCDR was not improved but slightly degraded, showing that such a subcomponent design does augment interpretability as well as facilitates prediction. Overall, SubCDR with the above five modules together delivered superior predictive performance, and removing any modules will compromise its predictive power.

## 4 Discussion

Predicting cancer drug response (CDR) is a major goal in cancer research for computer-aided anti-cancer drug discovery and precision medicine. In this work, we developed a subcomponent-guided deep learning model for interpretable CDR prediction, named SubCDR, which offers a novel view of understanding the biological significance behind predictions, in terms of subcomponent interactions between drugs and cell lines. The predictive ability of SubCDR has been validated on the GDSC dataset and compared with state-of-the-art methods under various evaluation settings. Extensive case studies have exhibited the capacity of SubCDR in interpreting predictions and finding the relevant subcomponents driving response outcomes.

Despite our efforts, there is still room for improvement. The performance of SubCDR falls short of expectations when predicting CDRs for unseen drugs and cell lines. In such cold start issues, there is no prior knowledge about unseen drugs/cell lines, and SubCDR can only infer responses based on the similarity of feature spaces between seen and unseen drugs/cell lines. Inspired by the knowledge graph (KG) on the biomedicine field [56], incorporating additional domain knowledge associated with unseen drugs/cell lines, e,g., drug-target affinities, target pathways or gene regulatory networks, into our framework has the potential to mitigate this issue. Besides, the interpretability of SubCDR is expected to be continuously extended, especially in the design and extraction of subcomponents. For the cell line, there are two alternatives: (1) subcomponents could be defined according to positions and types of gene mutations, to identify the role of major cancer-causing mutations in CDR predictions, (2) subcomponents could be implemented as biological pathways (i.e., gene sets involved in a given pathway that is coordinately up- or down-regulated), to show how pathway signatures derived from cell lines work to CDR predictions. For the drug, diversified subcomponent forms, including but not limited to fingerprint fragments [57] and molecular motifs [58], could also be implanted into our framework with appropriate modifications.

## Supporting information

S1 Text (a) Classification of CGC genes in different cancer cell lines. (b) Implementation of the baseline methods.
(DOCX)

S1 Fig The receiver operating characteristic (ROC) and precision-recall (PR) curve of SubCDR and baseline methods in the classification task of independent testing. (TIF)

S2 Fig PCC (up) and R^2^ (down) scores of all methods across the different cancer types (defined in the TCGA study) of cell lines and target pathway types of drugs. (TIF)

S3 Fig AUC (up) and AUPR (down) scores of all methods across the different cancer types (defined in the TCGA study) of cell lines and target pathway types of drugs. Of note, AUC/AUPR scores cannot be calculated for some target pathway group data, because they do not include instances of positive (sensitive) labels, i.e., ln(IC_50_) values are all greater than -2.
(TIF)

S4 Fig (a) Cases of drug Gefitinib’s response to cell line NCI-H1650 and NCI-H1568. (b) Cases of drug Dactolisib’s response to cell line KARPAS-45 and P30-OHK. (c) Cases of drug Cytarabine and Gemcitabine responding to cell lines HCC78, NCI-H1792, and NCI-H1993, respectively.
(TIF)

S1 Table (a) Annotation information for drugs. (b) Annotation information for cell lines.
(DOCX)
