# Supplementary material for: A subcomponent-guided deep learning method for interpretable cancer drug response prediction
Source: PLoS Comput Biol. 2023 Aug 21;19(8):e1011382. doi: 10.1371/journal.pcbi.1011382 (PMC10470940; doi:10.1371/journal.pcbi.1011382)
Supplement: S1 Text — (a) Classification of CGC genes in different cancer cell lines. (b) Implementation of the baseline methods. (DOCX) [file pcbi.1011382.s001.docx]

**Supporting information——S1 Tex**

1. **Classification of CGC genes in different cancer cell lines**

The CGC project of COSMIC (https://cancer.sanger.ac.uk/census) provides relevant (previous literature reported) tumour types for each cancer-driving gene. Herein, we rely on the tumour or tissue types of cancer cell lines provided by the CellModelPassports hub (https://cellmodelpassports.sanger.ac.uk/downloads), to match each cell line with specific cancer-driving genes. Organized annotation information about the CGC project and the CellModelPassports hub, and their matching results are provided at https://github.com/liuxuan666/SubCDR/tree/main/data/masked_cancer_gene.

1. **Implementation of the baseline methods**

All the baseline methods were implemented by their publicly available source codes. The optimal or default hyperparameters of each baseline method were deployed under our dataset and experimental settings. Notably, there is one method (i.e., GraphCDR) that has not been applied to the regression task and one method (i.e., BiG-DRP) that has not been applied to the classification task, in their original designs. Therefore, we carefully modified GraphCDR by removing its Sigmoid activation function at the last layer, and replacing the binary cross entropy (BCE) loss function with mean squared error, to ensure that it can be suitably applied to the regression task. For the BiG-DRP, we complemented the classification task by adding a Sigmoid activation function at the last layer and taking the BCE as the loss function during the training phase. Hyperparameters of our model and baselines are tuned to perform their best results (RMSE scores) on the warm start scenario of the regression task, and then fixed on the independent testing and classification tasks.
