# Supplementary figures and images for: A subcomponent-guided deep learning method for interpretable cancer drug response prediction
Source: PLoS Comput Biol. 2023 Aug 21;19(8):e1011382. doi: 10.1371/journal.pcbi.1011382 (PMC10470940; doi:10.1371/journal.pcbi.1011382)

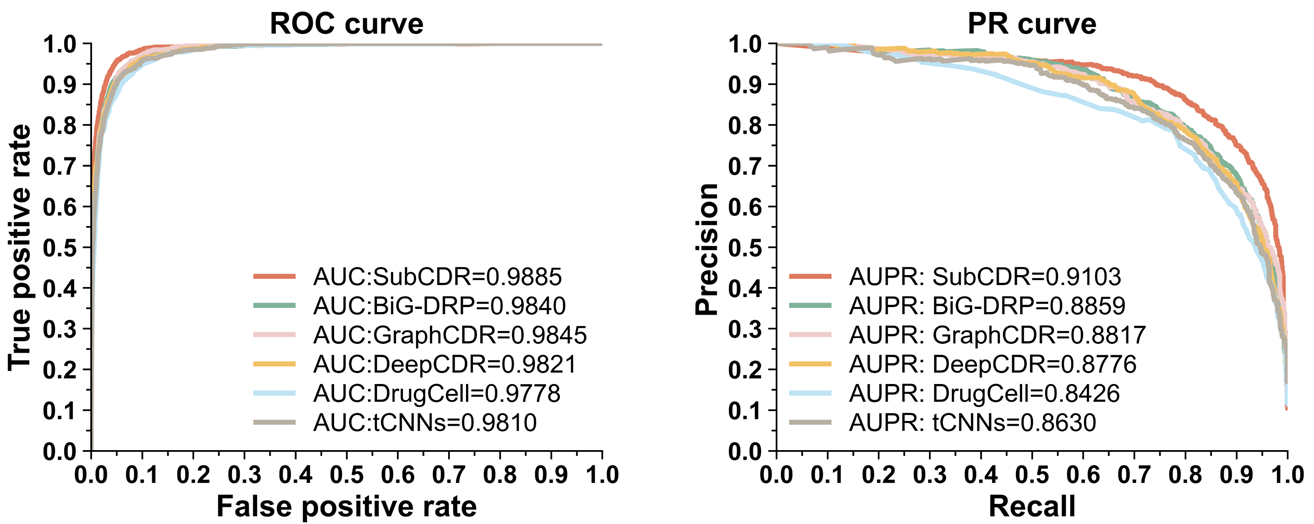

Supplement: S1 Fig — (TIF) [file pcbi.1011382.s002.tif]

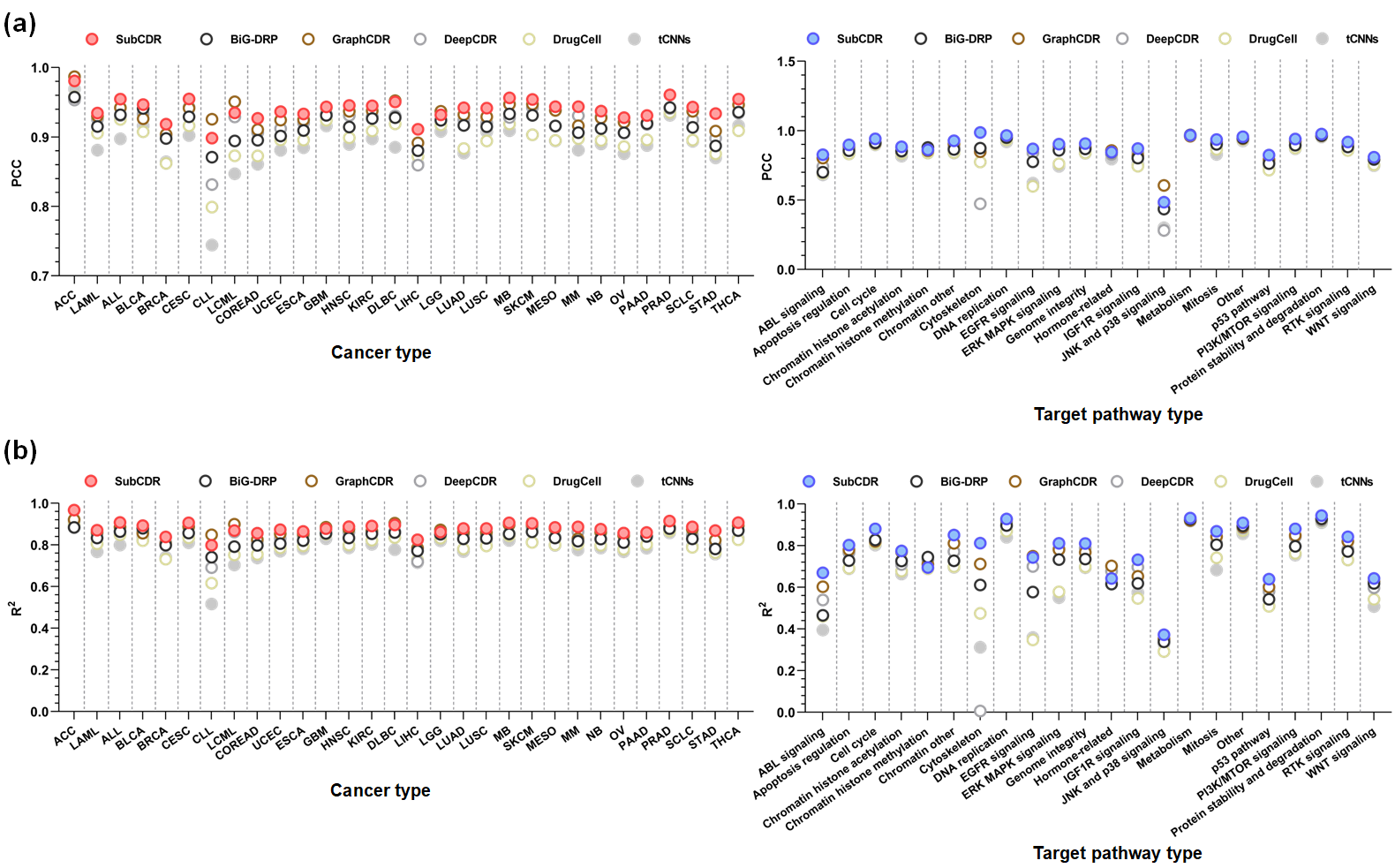

Supplement: S2 Fig — (TIF) [file pcbi.1011382.s003.tif]

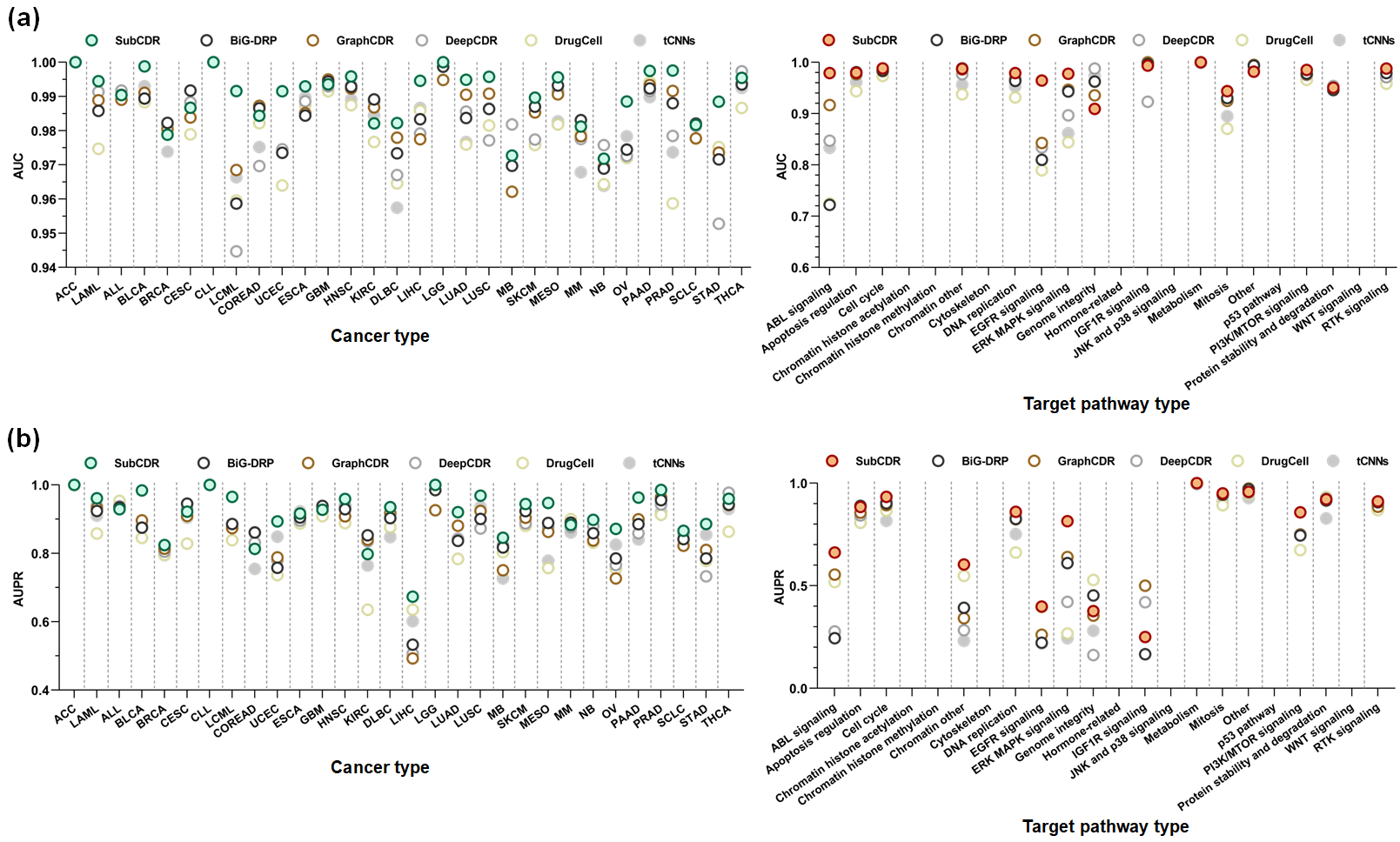

Supplement: S3 Fig — AUC (up) and AUPR (down) scores of all methods across the different cancer types (defined in the TCGA study) of cell lines and target pathway types of drugs. Of note, AUC/AUPR scores cannot be calculated for some target pathway group data, because they do not include instances of positive (sensitive) labels, i.e., ln(IC50) values are all greater than -2. (TIF) [file pcbi.1011382.s004.tif]

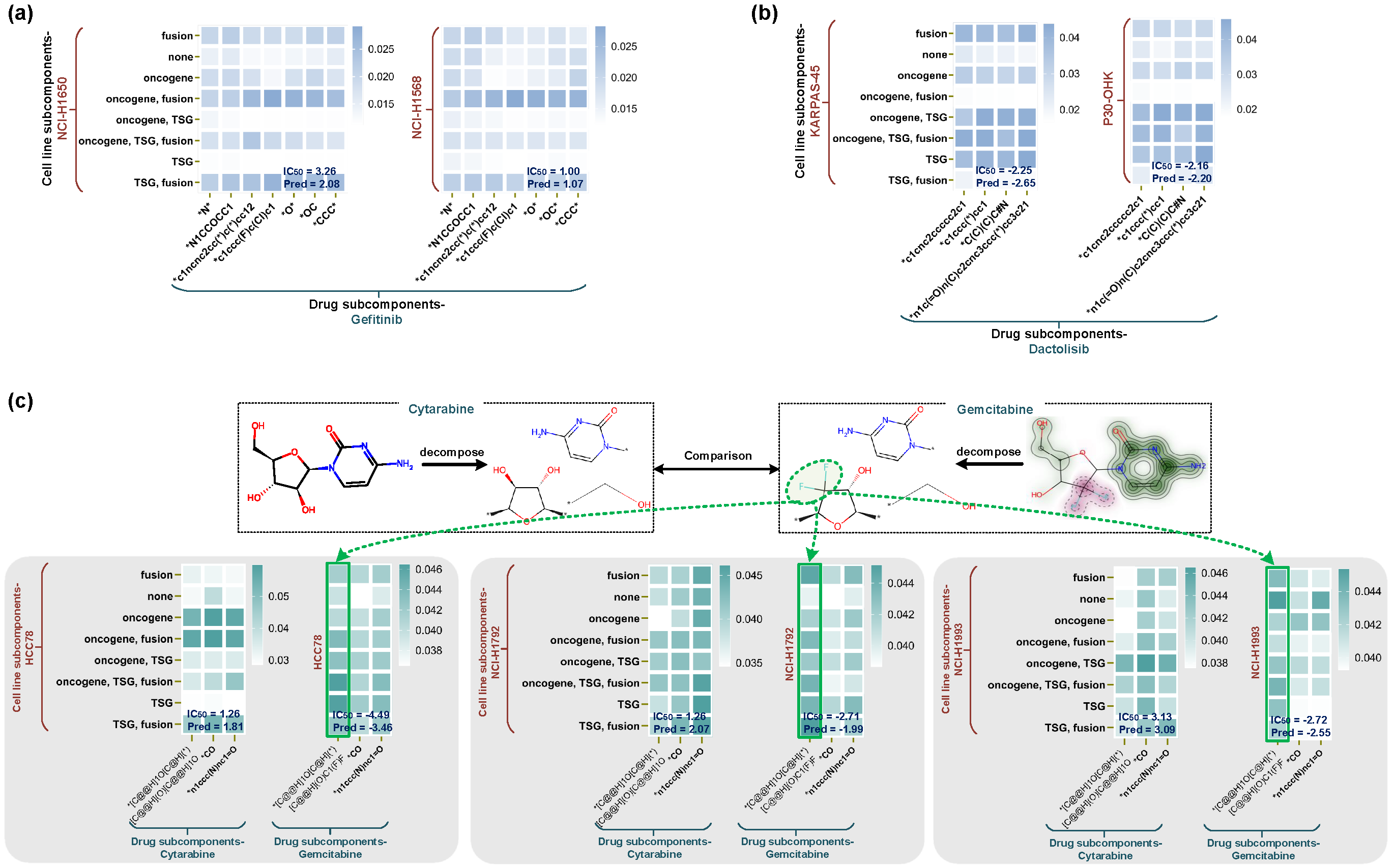

Supplement: S4 Fig — (a) Cases of drug Gefitinib’s response to cell line NCI-H1650 and NCI-H1568. (b) Cases of drug Dactolisib’s response to cell line KARPAS-45 and P30-OHK. (c) Cases of drug Cytarabine and Gemcitabine responding to cell lines HCC78, NCI-H1792, and NCI-H1993, respectively. (TIF) [file pcbi.1011382.s005.tif]
