# Supplementary material for: A subcomponent-guided deep learning method for interpretable cancer drug response prediction
Source: PLoS Comput Biol. 2023 Aug 21;19(8):e1011382. doi: 10.1371/journal.pcbi.1011382 (PMC10470940; doi:10.1371/journal.pcbi.1011382)
Supplement: S1 Table — (a) Annotation information for drugs. (b) Annotation information for cell lines. (DOCX) [file pcbi.1011382.s006.docx]

**Supporting information——S1 Table**

1. **Annotation information for drugs**

| **Pubchem ID** | **Drug name** | **Target pathway** | **SMILES string** |
| --- | --- | --- | --- |
| 462382 | MG-132 | Protein stability and degradation | CC(C)C[C@@H](C=O)NC(=O)[C@H](CC(C)C)NC(=O)[C@H](CC(C)C)NC(=O)OCC1=CC=CC=C1 |
| 16654980 | Buparlisib | PI3K/MTOR signaling | C1COCCN1C2=NC(=NC(=C2)C3=CN=C(C=C3C(F)(F)F)N)N4CCOCC4 |
| 11719003 | Ulixertinib | ERK MAPK signaling | CC(C)NC1=NC=C(C(=C1)C2=CNC(=C2)C(=O)N[C@H](CO)C3=CC(=CC=C3)Cl)Cl |
| 3385 | 5-Fluorouracil | Other | C1=C(C(=O)NC(=O)N1)F |
| 3062316 | Dasatinib | Other, kinases | CC1=C(C(=CC=C1)Cl)NC(=O)C2=CN=C(S2)NC3=CC(=NC(=N3)C)N4CCN(CC4)CCO |
| 46843057 | Afuresertib | PI3K/MTOR signaling | CN1C(=C(C=N1)Cl)C2=C(SC(=C2)C(=O)N[C@@H](CC3=CC(=CC=C3)F)CN)Cl |
| 56645356 | AGI-5198 | Metabolism | CC1=CC=CC=C1C(C(=O)NC2CCCCC2)N(C3=CC(=CC=C3)F)C(=O)CN4C=CN=C4C |
| 78209992 | AZD3759 | EGFR signaling | C[C@@H]1CN(CCN1C(=O)OC2=C(C=C3C(=C2)C(=NC=N3)NC4=C(C(=CC=C4)Cl)F)OC)C |
| 25227436 | AZD5363 | Other, kinases | C1CN(CCC1(C(=O)N[C@@H](CCO)C2=CC=C(C=C2)Cl)N)C3=NC=NC4=C3C=CN4 |
| 54761306 | AZD6738 | Genome integrity | C[C@@H]1COCCN1C2=NC(=NC(=C2)C3(CC3)[S@](=N)(=O)C)C4=C5C=CNC5=NC=C4 |
| 52913813 | AZD8186 | PI3K/MTOR signaling | C[C@H](C1=CC(=CC2=C1OC(=CC2=O)N3CCOCC3)C(=O)N(C)C)NC4=CC(=CC(=C4)F)F |
| 71496458 | Osimertinib | EGFR signaling | CN1C=C(C2=CC=CC=C21)C3=NC(=NC=C3)NC4=C(C=C(C(=C4)NC(=O)C=C)N(C)CCN(C)C)OC |
| 9933475 | Cediranib | RTK signaling | CC1=CC2=C(N1)C=CC(=C2F)OC3=NC=NC4=CC(=C(C=C43)OC)OCCCN5CCCC5 |
| 24788740 | Ipatasertib | PI3K/MTOR signaling | C[C@@H]1C[C@H](C2=C1C(=NC=N2)N3CCN(CC3)C(=O)[C@H](CNC(C)C)C4=CC=C(C=C4)Cl)O |
| 56941241 | GDC0810 | Hormone-related | CC/C(=C(/C1=CC=C(C=C1)/C=C/C(=O)O)\\C2=CC3=C(C=C2)NN=C3)/C4=C(C=C(C=C4)F)Cl |
| 70676303 | GNE-317 | PI3K/MTOR signaling | CC1=C(SC2=C1N=C(N=C2N3CCOCC3)C4=CN=C(N=C4)N)C5(COC5)OC |
| 5394 | Temozolomide | DNA replication | CN1C(=O)N2C=NC(=C2N=N1)C(=O)N |
| 16747683 | AZD5438 | Cell cycle | CC1=NC=C(N1C(C)C)C2=NC(=NC=C2)NC3=CC=C(C=C3)S(=O)(=O)C |
| 68107965 | GSK2578215A | Other, kinases | C1=CC=C(C=C1)COC2=C(C=C(C=C2)C3=CC(=NC=C3)F)C(=O)NC4=CN=CC=C4 |
| 91668541 | I-BRD9 | Chromatin other | CCN1C=C(C2=C(C1=O)C=C(S2)C(=NC3CCS(=O)(=O)CC3)N)C4=CC(=CC=C4)C(F)(F)F |
| 10385095 | Telomerase Inhibitor IX | Genome integrity | C1=CC(=CC(=C1)NC(=O)C2=C(C(=CC=C2)O)O)NC(=O)C3=C(C(=CC=C3)O)O |
| 227681 | MIRA-1 | p53 pathway | CCC(=O)OCN1C(=O)C=CC1=O |
| 9825149 | NVP-ADW742 | IGF1R signaling | C1CCN(C1)CC2CC(C2)N3C=C(C4=C(N=CN=C43)N)C5=CC(=CC=C5)OCC6=CC=CC=C6 |
| 46931953 | P22077 | Protein stability and degradation | CC(=O)C1=CC(=C(S1)SC2=C(C=C(C=C2)F)F)[N+](=O)[O-] |
| 68289010 | Savolitinib | RTK signaling | C[C@@H](C1=CN2C=CN=C2C=C1)N3C4=NC(=CN=C4N=N3)C5=CN(N=C5)C |
| 992586 | UMI-77 | Apoptosis regulation | C1=CC=C2C(=C1)C(=CC(=C2O)SCC(=O)O)NS(=O)(=O)C3=CC=C(C=C3)Br |
| 2984337 | WIKI4 | WNT signaling | COC1=CC=C(C=C1)N2C(=NN=C2SCCCN3C(=O)C4=CC=CC5=C4C(=CC=C5)C3=O)C6=CC=NC=C6 |
| 11178236 | Sepantronium bromide | Apoptosis regulation | CC1=[N+](C2=C(N1CCOC)C(=O)C3=CC=CC=C3C2=O)CC4=NC=CN=C4.[Br-] |
| 71297207 | WEHI-539 | Apoptosis regulation | C1CC2=C(C=C(C=C2)C3=NC(=C(S3)CCCOC4=CC=C(C=C4)CN)C(=O)O)/C(=N/NC5=NC6=CC=CC=C6S5)/C1 |
| 42642645 | Foretinib | RTK signaling | COC1=CC2=C(C=CN=C2C=C1OCCCN3CCOCC3)OC4=C(C=C(C=C4)NC(=O)C5(CC5)C(=O)NC6=CC=C(C=C6)F)F |

Note: Here, we present only 30 examples, and annotation information for all drugs is provided at https://github.com/liuxuan666/SubCDR/tree/main/data/.

1. **Annotation information for cell lines**

| **COSMIC ID** | **Cell line name** | **Cancer type defined in TCGA** | **Abbreviation for type** |
| --- | --- | --- | --- |
| 724834 | NCI-H2087 | Lung Adenocarcinoma | LUAD |
| 753615 | U-266 | Multiple myeloma | MM |
| 753618 | KELLY | Neuroblastoma | NB |
| 724838 | UM-UC-3 | Bladder Cancer | BLCA |
| 724866 | NCI-H1355 | Lung Adenocarcinoma | LUAD |
| 724868 | NCI-H1792 | Lung Adenocarcinoma | LUAD |
| 724869 | HPAF-II | Pancreatic Cancer | PAAD |
| 724870 | MIA-PaCa-2 | Pancreatic Cancer | PAAD |
| 724872 | SHP-77 | Small cell lung cancers | SCLC |
| 724873 | NCI-H2009 | Lung Adenocarcinoma | LUAD |
| 724879 | SW900 | Lung Squamous Cell Carcinoma | LUSC |
| 735784 | TE-5 | Esophageal Cancer | ESCA |
| 749712 | HCC1395 | Breast Cancer | BRCA |
| 749717 | HCC38 | Breast Cancer | BRCA |
| 753538 | Ca9-22 | Head and Neck Cancer | HNSC |
| 753547 | CPC-N | Small cell lung cancers | SCLC |
| 753552 | DSH1 | Bladder Cancer | BLCA |
| 753561 | HO-1-u-1 | Head and Neck Cancer | HNSC |
| 753562 | HSC-2 | Head and Neck Cancer | HNSC |
| 753563 | IM-9 | Multiple myeloma | MM |
| 753569 | KNS-62 | Lung Squamous Cell Carcinoma | LUSC |
| 753572 | KP-4 | Pancreatic Cancer | PAAD |
| 753576 | KYSE-70 | Esophageal Cancer | ESCA |
| 753584 | LB831-BLC | Bladder Cancer | BLCA |
| 753589 | LU-165 | Small cell lung cancers | SCLC |
| 753594 | MS-1 | Small cell lung cancers | SCLC |
| 753608 | PC-14 | Lung Adenocarcinoma | LUAD |
| 753610 | SBC-3 | Small cell lung cancers | SCLC |
| 753612 | SK-MM-2 | Multiple myeloma | MM |
| 753614 | TE-15 | Esophageal Cancer | ESCA |

Note: Here, we present only 30 examples, and annotation information for all cell lines is provided at https://github.com/liuxuan666/SubCDR/tree/main/data/.
